# Adverse childhood experiences and cyber aggression among college students: the mediating role of psychological resilience and the moderating role of physical exercise

**DOI:** 10.3389/fpsyt.2026.1943434

**Published:** 2026-09-24

**Authors:** Luyao Xiang, Hao Gou, Qunqun Sun, Faxiang Fan, Chang Hu

**Affiliations:** 1 Basic Teaching Department, Zunyi Medical University, Zhuhai, China; 2 College of Physical Education, Qiannan Normal University for Nationalities, Duyun, China; 3 College of Physical Education, Hunan First Normal University, Changsha, China

**Keywords:** adverse childhood experiences, cumulative risk, cyber aggression, physical exercise, psychological resilience

## Abstract

**Background:**

To examine the association between adverse childhood experiences (ACEs) and cyber aggression (CA) among college students and to investigate the statistical mediating role of psychological resilience (PR) and the moderating role of physical exercise (PE).

**Methods:**

A cross-sectional questionnaire survey was conducted among 3,466 full-time Chinese college students. CA, childhood maltreatment, PR, and PE were assessed using the Adolescent Online Aggressive Behavior Scale, the Childhood Trauma Questionnaire–Short Form, the Connor–Davidson Resilience Scale, and the Physical Activity Rating Scale-3, respectively. The cumulative ACE count was used for polynomial regression and group comparisons, whereas the continuous ACE score was entered as X in PROCESS Models 4 and 14 to test the statistical indirect effect through PR and the moderating role of PE, respectively.

**Results:**

The cumulative ACE count showed significant positive linear (*β* = 0.438, *p* < 0.001) and quadratic (*β* = 0.520, *p* < 0.001) associations with CA, supporting an overall upward-curving association. Descriptive group means increased across the six cumulative-exposure groups. Using the continuous ACE score, PR statistically mediated the association between the ACE score and CA (indirect effect = 0.037). PE moderated the association between PR and CA (interaction coefficient = −0.011), with a stronger inverse PR–CA association at higher levels of PE.

**Conclusion:**

A higher cumulative ACE count was associated with higher levels of CA, and the significant positive quadratic term supported an overall upward-curving association. Higher continuous ACE scores were indirectly associated with higher CA through lower PR, while PE strengthened the inverse association between PR and CA. Given the cross-sectional design, these findings should be interpreted as statistical associations rather than causal relationships.

## 1 Introduction

According to the 54th Statistical Report on Internet Development in China, the number of Internet users in China has reached 1.079 billion, with an Internet penetration rate of 76.4%, indicating that a digital society has largely taken shape (1). While technological advances have substantially improved convenience and efficiency in everyday life (2), the online disinhibition effect has also contributed to various forms of behavioral misconduct in cyberspace, including deviant behaviors that violate or undermine established online norms (3). Cyber aggression (CA) is a representative form of such misconduct and refers to the deliberate infliction of harm on others through information and communication technologies (4). As digital natives, college students are particularly active in online environments, and the prevalence of CA within this population has continued to increase (5). Studies of Chinese university students have documented cyberbullying perpetration across different samples and measurement approaches (6, 7); in one study, 25.98% of participants reported bullying others online during the semester (6). Compared with traditional aggression, CA is characterized by virtuality, anonymity, and freedom from temporal and spatial constraints, making it more difficult to regulate effectively and potentially more harmful to those involved (8). Cyberbullying involvement has also been associated with poorer psychological well-being and suicide ideation/behavior in student populations (9). Therefore, a deeper understanding of the mechanisms underlying CA among college students is essential for providing a sound theoretical basis for its effective prevention and intervention.

### 1.1 Adverse childhood experiences and cyber aggression

Adverse childhood experiences (ACEs) are important correlates of aggressive behavior and may have profound implications for adolescent development (10, 11). ACEs conventionally refer to potentially traumatic experiences occurring before the age of 18, including abuse, neglect, and household dysfunction (12). The present study focused on five childhood maltreatment domains: emotional abuse, physical abuse, sexual abuse, emotional neglect, and physical neglect. International evidence indicates that ACEs are highly prevalent among adolescents. For example, Carlson et al. conducted a systematic review of studies published between 1990 and 2015 and found that approximately two-thirds of adolescents worldwide had experienced at least one major adverse event (13). Among college students, 40% of respondents reported exposure to at least one ACE, including 11% who reported abuse, 14% who reported family dysfunction, and 17% who reported both (14, 15). Previous research among college students has shown that childhood maltreatment is positively associated with cyberbullying, with hostile attribution bias and anger rumination implicated as cognitive processes in this association (16). From a theoretical perspective, attachment theory proposes that childhood trauma may impair emotion regulation and empathy, contribute to insecure attachment patterns, and subsequently increase hostility and aggression in interpersonal interactions (17). Social learning theory, by contrast, emphasizes the role of observational learning in the intergenerational transmission of aggression, suggesting that exposure to parental aggression may increase the likelihood of aggressive behavior in children (18).

Existing research on ACEs has largely focused on the linear associations of individual risk factors with adverse outcomes (19, 20), whereas the cumulative risk model emphasizes that the combined influence of multiple risks may produce nonlinear behavioral consequences (21). Previous studies have shown that early exposure to even a single category of adverse experience is associated with a substantially higher prevalence of aggressive behavior in online contexts during young adulthood (22, 23). Further evidence indicates that different categories of ACEs frequently co-occur, such that individuals may experience multiple forms of childhood trauma simultaneously (24). According to the cumulative risk model, cumulative ACE exposure can be classified into two levels. Low cumulative ACE exposure refers to experiencing one or two of the five categories of ACEs—emotional abuse, physical abuse, sexual abuse, emotional neglect, and physical neglect—whereas high cumulative ACE exposure refers to experiencing three or more categories (25). Cross-sectional research has shown that, among individuals in higher education, 38.2% report low cumulative ACE exposure and 15.4% report high cumulative exposure (26). Adverse experiences encountered during childhood may therefore exert cumulative and compounding influences on adolescent development (27). Compared with any single risk factor, the combined burden of multiple adverse experiences appears to be more strongly associated with developmental outcomes among college students (21).

Within the theoretical framework of the cumulative risk model, the association between ACEs and CA may follow three typical patterns, as illustrated in **Figure 1**. First, the linear model proposes a significant positive association between cumulative ACE count and the prevalence of CA, consistent with the simple additive-risk hypothesis (21). Second, the increasing marginal effects model proposes that, once cumulative ACE exposure reaches a critical level, the association of each additional risk factor with CA increases exponentially. Third, the risk saturation threshold model proposes that, as cumulative risk continues to increase, the incremental association of each additional risk factor with CA gradually weakens, a pattern theoretically consistent with resource-allocation theory in behavioral ecology (28). Recent evidence has identified nonlinear associations between cumulative ACE exposure and several youth behavioral outcomes (29). Whether a comparable nonlinear pattern characterizes the association between cumulative ACE exposure and CA among college students remains unclear. Accordingly, the present study adopted the cumulative risk model as its primary theoretical framework. Based on the model’s central proposition that exposure to a greater number of risk factors is associated with a disproportionately higher likelihood of adverse outcomes (30), we hypothesized that cumulative ACE count would show positive linear and quadratic associations with CA, consistent with an upward-curving nonlinear pattern.

**Figure 1:**
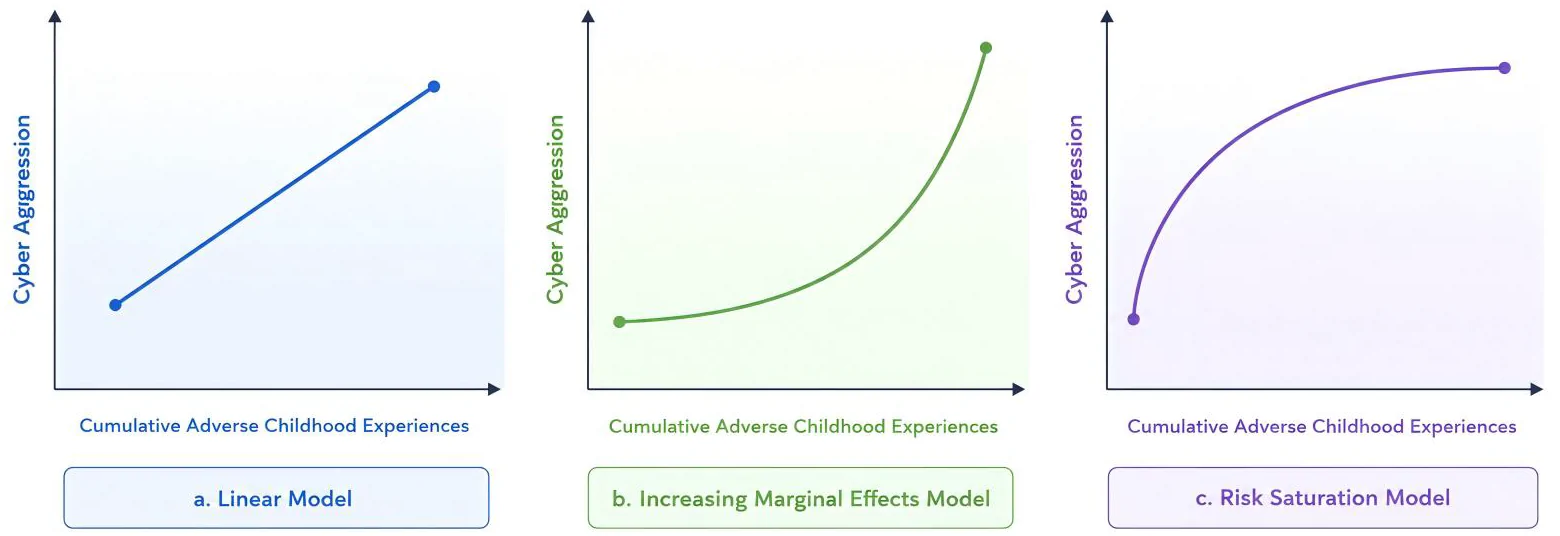
Three typical patterns of association between cumulative ACEs and cyber aggression.

### 1.2 Psychological resilience as a statistical mediator

Further clarification of the psychological pathways linking ACEs and CA among college students represents another important issue in this field (31). The Intergenerational and Cumulative Adverse and Resilient Experiences (ICARE) model proposes that the developmental consequences associated with ACEs partly reflect biobehavioral adaptations to early-life stress (32). These adaptations may alter cognitive, social, and emotional development, while their adverse effects may be mitigated by psychologically protective relationships and contextual resources (33, 34). Psychological resilience (PR) refers to an individual’s capacity to respond adaptively to adversity, trauma, or stress by mobilizing personal resources to recover from and grow through challenging experiences (35). In the present framework, PR is conceptualized as an intervening psychological resource in the statistical association between ACEs and CA. Mediation therefore refers to an indirect association in which ACE exposure is associated with PR and PR is, in turn, associated with CA, whereas moderation would imply that the magnitude of the ACE–CA association differs across levels of PR. University evidence supports both component associations. Sezgin reported a negative association between ACE exposure and resilience among female university students, while Chen et al. found that childhood emotional maltreatment was negatively associated with resilience and that resilience partially mediated its association with aggression among Chinese college students (36, 37). Direct evidence specific to CA remains limited in college populations, but adolescent studies have shown that psychological resilience statistically mediated the association between childhood psychological abuse and cyberbullying and was negatively associated with cyberbullying perpetration (38, 39).

### 1.3 Physical exercise as a moderator

Physical exercise (PE) has been widely regarded as an important means of enhancing PR, and its potential moderating role in the association between PR and aggressive behavior has received theoretical support (27, 40, 41). Empirical evidence further suggests that physical activity is positively associated with self-efficacy and self-control and negatively associated with aggressive behavior among college students, providing a behavioral basis for examining whether PE may strengthen the protective association between PR and CA (42, 43). For example, research among college students suggests that regular PE may be associated with higher levels of PR and lower levels of childhood trauma-related hostile attribution bias, thereby reducing the tendency to engage in aggressive behavior (44, 45). Further evidence suggests that the moderating role of PE may operate through the dynamic replenishment of resilience-related resources (46, 47). Among individuals with lower PR, PE may improve self-control and stress-coping capacity, thereby reducing the likelihood of aggressive responding when resilience resources are limited (48). Among those with higher PR, PE may further consolidate the protective function of resilience and contribute to a mutually reinforcing adaptive process (49). This mechanism suggests that PE may not operate solely through direct changes in emotional or cognitive functioning; rather, it may moderate the association between PR and CA by enhancing the functional effectiveness of resilience.

Based on the preceding theoretical arguments and review of the literature, the following hypotheses were proposed:

H1: A higher cumulative ACE count is associated with higher CA, with a positive quadratic component indicating an upward-curving nonlinear pattern.

H2: PR statistically mediates the association between the continuous ACE score and CA among college students.

H3: PE moderates the association between PR and CA among college students.

The present study tested these hypotheses using a moderated mediation model, as illustrated in **Figure 2**.

**Figure 2:**
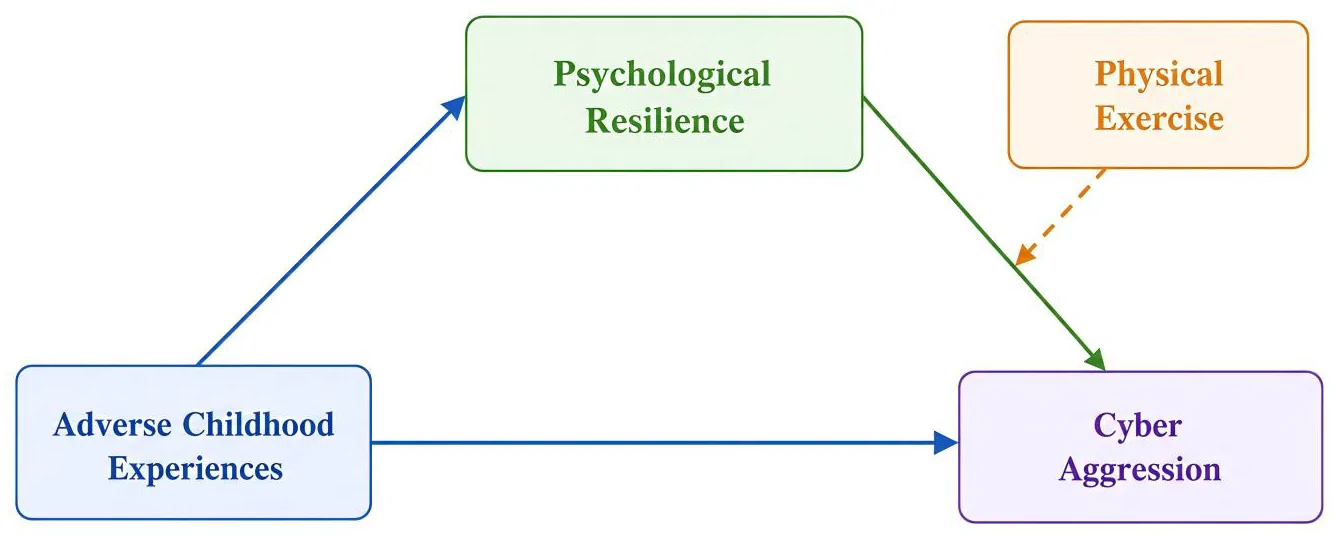
The hypothesized moderated mediation model.

## 2 Methods

### 2.1 Participants

A cross-sectional survey was conducted between September and December 2024 using purposive sampling. Universities located in Guizhou, Jiangxi, Guangdong, Hubei, Jiangsu, and Yunnan provinces were selected to reflect differences in geographic distribution and socioeconomic development. Within each province, participating institutions were selected according to university type, such as comprehensive and teacher-training universities, as well as the feasibility of collaboration with the research team, with the aim of enhancing sample diversity and the credibility of the findings. The final analytic sample of 3,466 students was recruited across these six provinces, with the recruitment frame including both comprehensive and teacher-training universities. Individual university names are not reported to avoid unnecessary institutional identification. Province- and university-level identifiers were not retained as analytic variables in the final de-identified dataset; therefore, verified post-screening sample sizes and proportions by province or university type are not available for reporting. The inclusion criteria were as follows: participants were required to be 18–25 years of age, enrolled as full-time undergraduate or postgraduate students, and willing to participate voluntarily. Questionnaires were excluded if the completion time was less than 5 minutes or more than 30 minutes, or if the responses showed clear patterns of repetitive or invariant responding.The survey was administered to students through Wenjuanxing, an online questionnaire platform. A pilot test was conducted before the formal survey to evaluate feasibility and applicability. Responses were screened using the prespecified completion-time and response-pattern criteria described above. Incomplete submissions and responses showing invariant or otherwise clearly anomalous patterns were excluded before analysis. A total of 3,600 questionnaires were collected. After 134 questionnaires were excluded because of patterned responding or other data-quality concerns, 3,466 valid questionnaires were retained, yielding a valid response rate of 96.3%. The final sample comprised 1,585 male and 1,881 female students. By year of study, 873 were first-year undergraduates, 1,029 were second-year undergraduates, 764 were third-year undergraduates, 538 were fourth-year undergraduates, and 262 were postgraduate students. Regarding household registration, 1,458 participants were from urban areas and 2,008 were from rural areas. The mean age of the participants was 21.20 years (SD = 1.50).

Ethics and Informed Consent: The study protocol was reviewed and approved by the relevant institutional ethics committee. All participants were adults aged 18 years or older and provided electronic informed consent on their own behalf before participation. Participation was voluntary, all responses were collected anonymously, and participants were free to withdraw at any time without penalty.

### 2.2 Measures

#### 2.2.1 Cyber aggression

CA was assessed using the Adolescent Online Aggressive Behavior Scale (AOABS) developed by Zhao and Gao (50). The AOABS consists of 31 items across two subscales: instrumental aggression (15 items) and reactive aggression (16 items). Each item is rated on a 4-point scale ranging from 1 (never) to 4 (always). The mean item score was used in the analyses, with higher scores indicating higher levels of CA. The AOABS has been widely used to assess cyber-aggressive behavior in Chinese student populations.

In the present study, the AOABS demonstrated good internal consistency, with a Cronbach’s α of 0.894. Confirmatory factor analysis of the two-factor structure indicated good model fit: *χ²/df* = 1.641, root mean square error of approximation (RMSEA) = 0.014, standardized root mean square residual (SRMR) = 0.012, comparative fit index (CFI) = 0.993, and Tucker–Lewis index (TLI) = 0.993.

#### 2.2.2 Adverse childhood experiences

ACEs were assessed using the Chinese version of the Childhood Trauma Questionnaire–Short Form (CTQ-SF), originally developed by Bernstein et al. (51) and translated into Chinese by Fu et al. (52). The CTQ-SF consists of 28 items, including 25 clinical items and three minimization/denial validity items. The clinical items are organized into five subscales, each containing five items: emotional abuse (EA), physical abuse (PA), sexual abuse (SA), emotional neglect (EN), and physical neglect (PN). The three minimization/denial items were not included in the calculation of the clinical score. In the present study, ACE exposure was assessed across these five CTQ-SF maltreatment domains. Each item is rated on a 5-point scale ranging from 1 (never true) to 5 (very often true). For analyses treating ACEs as a continuous variable, the mean of the 25 clinical items was calculated as the continuous ACE score, with higher scores indicating greater childhood maltreatment severity. Based on the cutoff criteria proposed by Bernstein et al., exposure to each ACE category was defined using the following thresholds: EA ≥ 13, PA ≥ 10, SA ≥ 8, EN ≥ 15, and PN ≥ 10. Each domain was coded as 0 when the cutoff was not met and 1 when the cutoff was met. The five binary domain scores were then summed to produce a cumulative ACE count ranging from 0 to 5. Participants who met the cutoff for at least one domain were classified as having experienced at least one ACE category.

In the present study, the CTQ-SF demonstrated good internal consistency, with a Cronbach’s α of 0.915. Confirmatory factor analysis of the five-factor structure indicated good model fit: *χ²/df* = 1.962, RMSEA = 0.017, SRMR = 0.022, CFI = 0.995, and TLI = 0.995.

#### 2.2.3 Psychological resilience

PR was assessed using the Chinese version of the 25-item Connor–Davidson Resilience Scale (CD-RISC), originally developed by Connor and Davidson (53) and adapted into Chinese by Yu and Zhang (54). The Chinese version comprises three dimensions: tenacity, strength, and optimism. Items are rated on a 5-point Likert scale ranging from 1 (not true at all) to 5 (true all the time). The mean item score was used in the analyses, with higher scores indicating higher levels of PR. The use of a 1–5 response scale is consistent with the Chinese version developed by Yu and Zhang.

In the present study, the CD-RISC demonstrated excellent internal consistency, with a Cronbach’s α of 0.962. Confirmatory factor analysis of the three-factor structure indicated good model fit: χ²/df = 1.789, RMSEA = 0.015, SRMR = 0.020, CFI = 0.997, and TLI = 0.996.

#### 2.2.4 Physical exercise

PE was assessed using the Physical Activity Rating Scale-3 (PARS-3) revised by Liang (55). The PARS-3 consists of three items assessing exercise intensity, duration, and frequency, with each item rated on a 5-point scale. The PARS-3 has been widely used in studies involving Chinese populations and has demonstrated good applicability across different age groups (56–59). The exercise volume score was calculated using the following formula:


Exercise volume = exercise intensity × (exercise duration − 1) × exercise frequency

The total score ranges from 0 to 100, with higher scores indicating greater exercise volume. Scores of 19 or below indicate a low exercise volume, scores from 20 to 42 indicate a moderate exercise volume, and scores of 43 or above indicate a high exercise volume. The continuous PARS-3 total score was used as the moderator in the conditional process analysis.

In the present study, the PARS-3 demonstrated acceptable internal consistency, with a Cronbach’s α of 0.711.

### 2.3 Statistical analysis

Data were analyzed using IBM SPSS Statistics version 27.0 and the PROCESS macro developed by Hayes. Variables involved in the quadratic and interaction terms were mean-centered before analysis. The cumulative ACE count was entered hierarchically in the polynomial regression, with the mean-centered linear term entered at Step 1 and the squared term entered at Step 2; the change in explained variance (ΔR²) was examined to evaluate the incremental contribution of the quadratic term. The six cumulative-exposure groups were used descriptively to illustrate mean CA scores across exposure levels. The continuous ACE score was used in the bivariate correlation analysis and was entered as X in PROCESS Model 4 to test the statistical mediating role of PR. The same continuous ACE score was entered as X in PROCESS Model 14 to examine whether PE moderated the association between PR and CA within the proposed moderated mediation model. Indirect effects in PROCESS Model 4 and the conditional indirect effects and index of moderated mediation in PROCESS Model 14 were estimated using 5,000 bootstrap resamples. Percentile bootstrap 95% confidence intervals were used for inference, and an effect was considered statistically significant when the confidence interval did not include zero. Sex, age, academic year, and urban/rural household registration were included as covariates in PROCESS Models 4 and 14. All coefficients reported from PROCESS Models 4 and 14 are unstandardized regression coefficients (B).

## 3 Results

### 3.1 Assessment of common method bias

Harman’s single-factor test was conducted to assess potential common method bias. The results identified 11 factors with eigenvalues greater than 1, which collectively accounted for 60.4% of the total variance. The first factor explained 20.4% of the total variance, which was below the recommended threshold of 40%, suggesting that common method bias was not pronounced in the present study (60).

### 3.2 Descriptive statistics and correlations among the study variables

The means, standard deviations, and bivariate correlations for CA, the continuous ACE score, PR, and PE are presented in **Table 1**. CA was positively correlated with the continuous ACE score and negatively correlated with both PR and PE. The continuous ACE score was negatively correlated with PR and PE, whereas PE was positively correlated with PR.

**Table 1:** Means, standard deviations, and correlations among the study variables.

Variable	*M*	*SD*	1	2	3	4
1. Cyber aggression	2.321	0.812	1			
2. Continuous ACE score	2.453	0.763	0.438***	1		
3. Psychological resilience	3.222	1.060	−0.330***	−0.334***	1	
4. Physical exercise	28.007	29.568	−0.210***	−0.146***	0.067***	1

M, mean; SD, standard deviation. ***p < 0.001.

### 3.3 Cumulative adverse childhood experiences and cyber aggression among college students

To distinguish among the three patterns proposed within the cumulative risk framework, polynomial regression was conducted using the mean-centered cumulative count of CTQ-SF-defined maltreatment categories. As shown in **Table 2**, both the linear term (*β* = 0.438, *p* < 0.001) and the quadratic term (*β* = 0.520, *p* < 0.001) were positive and significant. The linear-only model explained 19.2% of the variance in CA (*R*² = 0.192). Adding the quadratic term increased the explained variance to 19.9% (*R*² = 0.199), representing a significant incremental contribution (*ΔR*² = 0.007, *ΔF*(1, 3463) = 30.496, *p* < 0.001). These findings indicate that CA increased as childhood maltreatment categories accumulated and that the fitted association followed an upward-curving pattern. The mean CA score also increased across the six cumulative-exposure groups, from 1.2208 in the zero-category group to 3.6854 in the five-category group (**Figure 3**). The adjacent group differences varied in magnitude across exposure levels. Accordingly, the positive quadratic term was interpreted as evidence of overall curvature rather than a discrete threshold. At the level of the fitted polynomial, this pattern remained more consistent with an increasing marginal effects pattern than with either a simple linear additive pattern or a risk saturation pattern. Group sizes for ACE counts 0–5 were 432 (12.46%), 611 (17.63%), 474 (13.68%), 815 (23.51%), 809 (23.34%), and 325 (9.38%), respectively.

**Table 2:** Association between cumulative adverse childhood experiences and cyber aggression.

Predictor	*β*	SE	Lower 95% CI	Upper 95% CI
Cumulative adverse childhood experiences	0.438***	0.015	0.408	0.468
Cumulative adverse childhood experiences²	0.520***	0.094	0.335	0.704
R²	0.199			

***p < 0.001.

**Figure 3:**
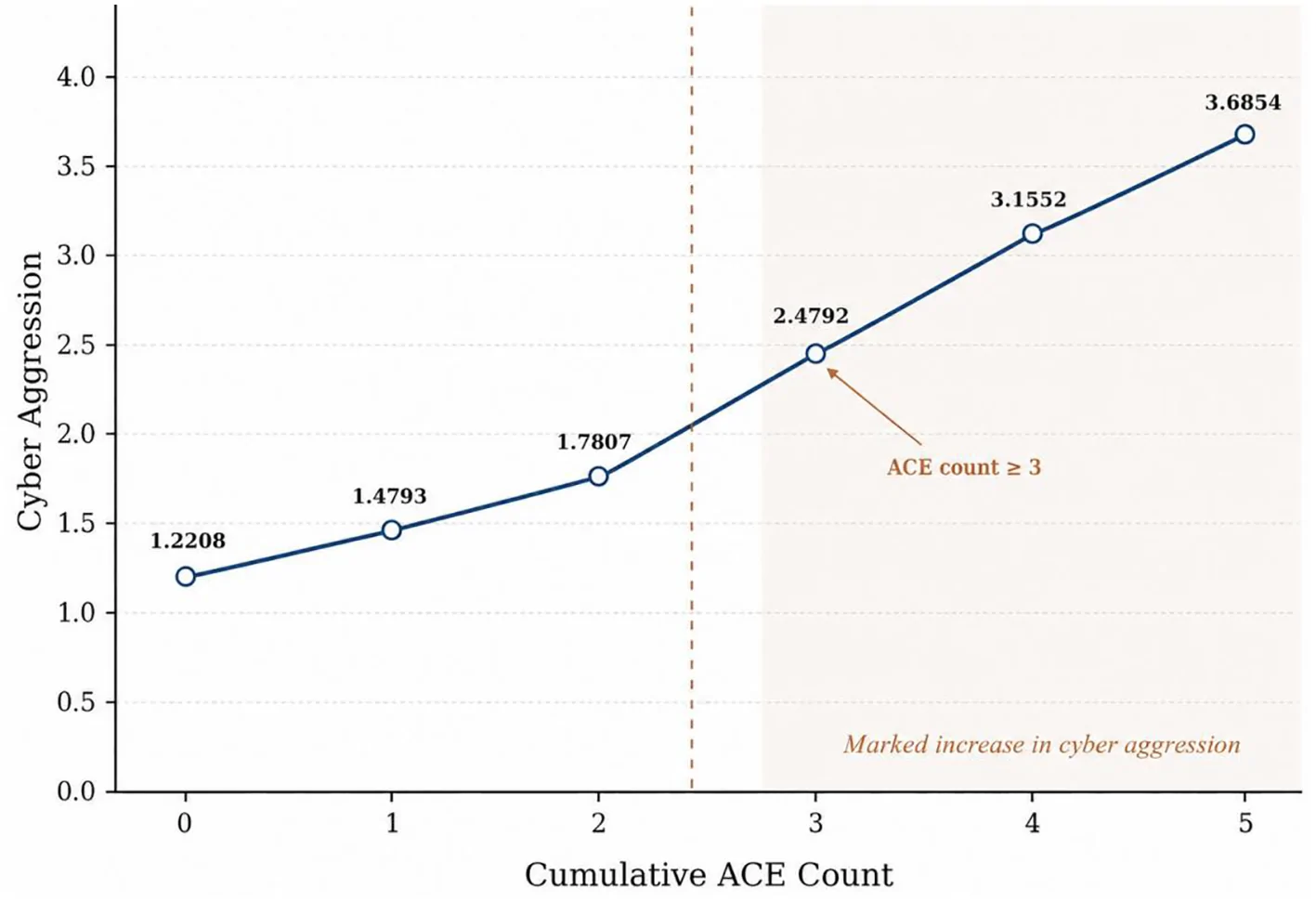
Mean cyber aggression scores across cumulative CTQ-SF-defined childhood maltreatment categories.

### 3.4 Statistical mediation analysis of psychological resilience

The mediating role of PR was examined using PROCESS version 4.1, Model 4, in SPSS Statistics 27.0. The continuous ACE score was specified as independent variable, CA as the dependent variable, and PR as the mediator. Sex, age, academic year, and urban/rural household registration were included as covariates. The continuous ACE score was significantly and negatively associated with PR (*B* = −0.459, *p* < 0.001), while PR was significantly and negatively associated with CA (*B* = −0.080, *p* < 0.001). After PR was included in the model, the continuous ACE score remained significantly and positively associated with CA (*B* = 0.196, *p* < 0.001). As shown in **Table 3**, bootstrap analysis indicated a significant indirect effect through PR of 0.037, with the 95% confidence interval excluding zero.

**Table 3:** Statistical mediating role of psychological resilience.

Model paths	Fit indices				Significance of coefficients			
	*R*	*R^2^*	*F*	*p*	*B*	(95%CI)		*p*
Continuous ACE score → Psychological Resilience	0.338	0.114	89.043	<0.001	−0.459	−0.503	−0.416	<0.001
Continuous ACE score → Cyber aggression	0.495	0.245	187.198	<0.001	0.196	0.180	0.213	<0.001
Psychological Resilience → Cyber aggression					−0.080	−0.092	−0.068	<0.001
Continuous ACE score → Psychological Resilience → Cyber aggression	0.454	0.206	179.929	<0.001	Total effect: 0.233	0.217	0.249	<0.001
					Indirect effect: 0.037	0.030	0.043	—

### 3.5 Moderating role of physical exercise

The moderating role of PE was examined using PROCESS version 4.1, Model 14, in SPSS Statistics 27.0, with the continuous ACE score entered as X. Sex, age, academic year, and urban/rural household registration were included as covariates. As shown in **Table 4**, the continuous ACE score was significantly and negatively associated with PR (*B* = −0.459, *p* < 0.001), and PR was significantly and negatively associated with CA (*B* = −0.093, p < 0.001). In addition, the interaction between PR and PE was significantly and negatively associated with CA (*B* = −0.011, *p* = 0.027), indicating that PE moderated the association between PR and CA.

**Table 4:** Moderating role of physical exercise.

Outcome	Predictor	*R*	*R²*	*F*	*B*	Lower 95% CI	Upper 95% CI	*t*
PR	Continuous ACE score	0.338	0.114	89.043	−0.459	−0.503	−0.416	−20.599
CA	Continuous ACE score	0.545	0.297	182.152	0.180	0.164	0.196	22.048
	PR				−0.093	−0.105	−0.082	−15.834
	PE				−0.080	−0.090	−0.070	−15.459
	PR × PE				−0.011	−0.021	−0.001	−2.211

To further examine this interaction, simple slope analyses were conducted at low and high levels of PE, defined as one standard deviation below and above the mean, respectively. As illustrated in **Figure 4**, PR was negatively associated with CA at both low (*B* = −0.081, *p* < 0.001) and high levels of PE (*B* = −0.106, *p* < 0.001). The stronger negative coefficient at the higher level of PE indicated that PE strengthened the inverse association between PR and CA.

**Figure 4:**
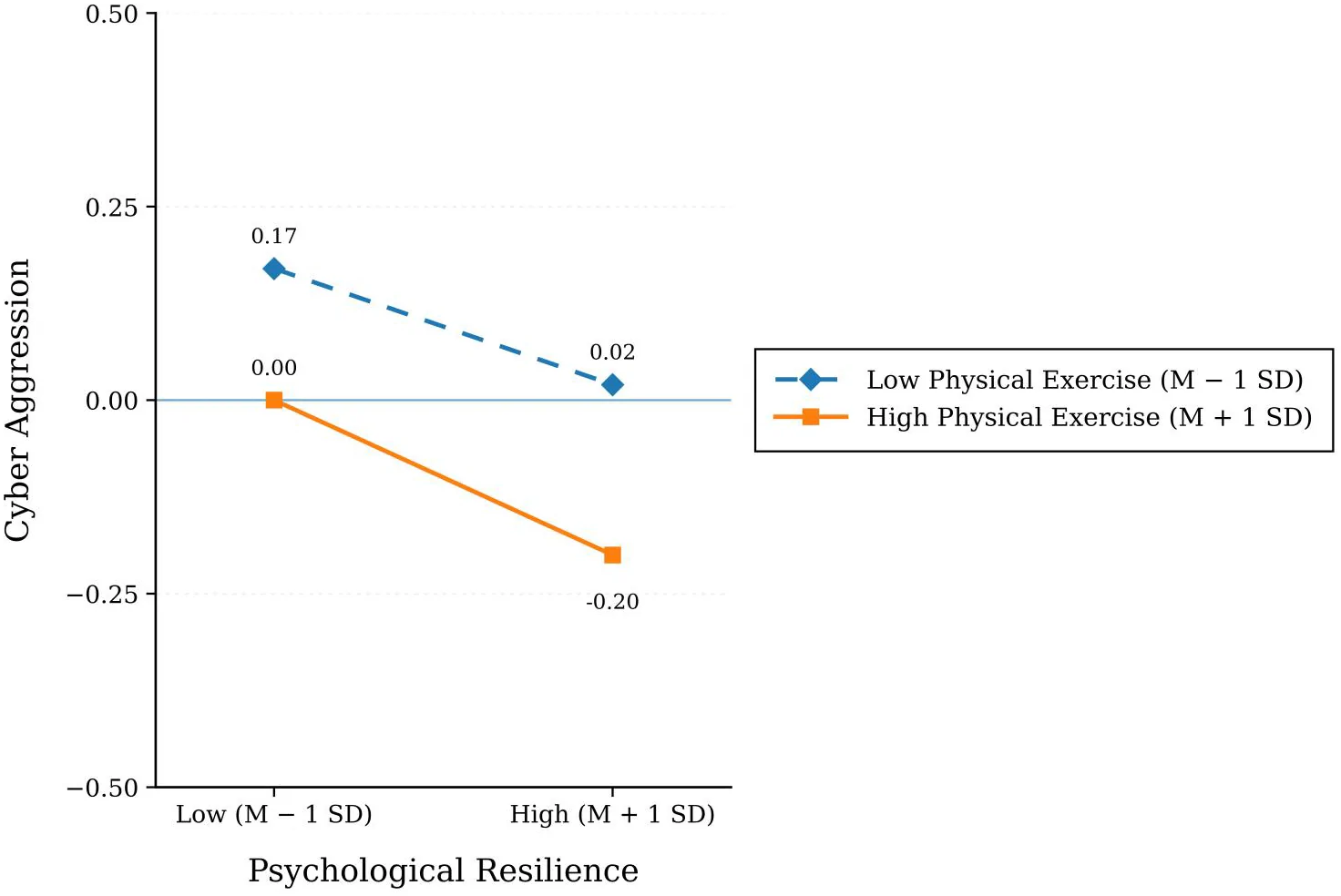
The moderating effect of physical exercise on the association between psychological resilience and cyber aggression.

As shown in **Table 5**, the conditional indirect effect of the continuous ACE score on CA through PR was 0.037. (BootSE = 0.005, 95% bootstrap CI [0.028, 0.046]) at low PE (M − 1 SD), 0.043 (BootSE = 0.003, 95% bootstrap CI [0.036, 0.050]) at the mean level of PE, and 0.049 (BootSE = 0.004, 95% bootstrap CI [0.041, 0.057]) at high PE (M + 1 SD). The index of moderated mediation was 0.0051 (BootSE = 0.0025, 95% bootstrap CI [0.0003, 0.0100]). Because the lower bound of the bootstrap confidence interval was greater than zero, the index remained statistically different from zero, indicating that the statistical indirect association through PR varied across levels of PE and was stronger at higher PE levels.

**Table 5:** Conditional indirect effects and index of moderated mediation.

Effect	PE level	Estimate	BootSE	BootLLCI	BootULCI
Indirect effect via PR	Low (M − 1 SD)	0.037	0.005	0.028	0.046
Indirect effect via PR	Mean (M)	0.043	0.003	0.036	0.050
Indirect effect via PR	High (M + 1 SD)	0.049	0.004	0.041	0.057
Index of moderated mediation	—	0.0051	0.0025	0.0003	0.0100

## 4 Discussion

Drawing on the cumulative risk model, the present study examined the associations among childhood maltreatment, PR, PE, and CA among college students. The results showed that CA increased with the cumulative ACE count, and the significant positive quadratic term supported an overall upward-curving association. Using the continuous ACE score, PR showed a significant statistical indirect effect in the association with CA, while PE moderated the association between PR and CA. These findings are consistent with an integrated framework of “risk accumulation–psychological resources–behavioral regulation” and highlight the potential value of resilience enhancement and exercise promotion in the prevention of CA among college students.

### 4.1 Association between adverse childhood experiences and cyber aggression among college students

The present study found that ACEs were positively associated with CA among college students, consistent with previous findings (61). Previous research has linked childhood psychological maltreatment with maladaptive interpersonal patterns and aggressive behavior (62). In college students, childhood maltreatment has also been associated with cyberbullying, with hostile attribution bias and anger rumination identified as relevant cognitive correlates (16). These findings are compatible with attachment theory, which emphasizes the role of early environmental experiences in later interpersonal functioning (17). Previous studies have further associated childhood maltreatment with cognitive difficulties (63) and hostile attribution bias in relation to reactive aggression (64). Together, this evidence offers plausible cognitive and interpersonal perspectives for interpreting the observed ACE–CA association.

Further examination of the association pattern suggested that the relationship between cumulative childhood maltreatment exposure and CA was more consistent with the increasing marginal effects model than with either a simple linear additive model or a risk saturation model. This interpretation is consistent with previous evidence showing that adverse outcomes may increase disproportionately at higher levels of cumulative ACE exposure. Merrick et al. (25) found that the risk of mental health problems increased markedly among individuals exposed to three or more ACE categories, while Hamby et al. (65) reported that the association between accumulated adversity and aggression became stronger at higher levels of exposure (66) Taken together, these findings support the cumulative risk proposition that the co-occurrence of multiple adverse experiences may be associated with disproportionately higher behavioral risk as exposure accumulates.

The upward-curving association may be interpreted in light of cumulative-risk perspectives proposing that co-occurring adversities place progressively greater demands on adaptive psychological resources (67). Prior studies have linked greater cumulative adversity to lower resilience-related resources (21, 68) and have suggested that different forms of adversity may jointly involve biological stress responses and cognitive-emotional processes, including hostile attribution bias (69, 70). Related work has used the concept of “trauma synergy” to describe the possibility that co-occurring adversities are associated with outcomes through overlapping or interacting processes (65). These perspectives offer possible explanations for the stronger fitted association at higher exposure levels. According to the cumulative risk model, the adverse associations of ACEs—including physical abuse, emotional neglect, and physical neglect—with physical and psychological well-being are not simply additive (71), and different forms of ACEs may be jointly associated with biological stress, cognitive development, and emotion-regulation processes (72). Longitudinal and mechanism-focused studies are needed to determine whether these processes account for the nonlinear pattern observed here. These findings further suggest that preventing or reducing exposure to any category of childhood adversity may be relevant to lowering the likelihood of externalizing problems, including CA (73).

### 4.2 Statistical mediating role of psychological resilience

Using the continuous ACE score, PR showed a significant statistical indirect effect in its association with CA among college students. Specifically, higher continuous ACE scores were associated with lower PR, and lower PR was in turn associated with higher CA. The ICARE model conceptualizes ACE-related outcomes as reflecting biobehavioral adaptations to early-life stress that may influence development and health, while emphasizing the importance of protective relationships and contextual resources in mitigating these adverse associations (33). This pattern is consistent with mediation: PR was modeled as an intervening correlate through which part of the ACE–CA association was statistically represented, rather than as a variable that altered the strength of the ACE–CA association. Within the ICARE framework, early adversity may be associated with changes in adaptive psychological resources, while resilience reflects the capacity to regulate and recover under stress (33). Lower PR may therefore coincide with greater difficulty managing negative affect and maladaptive responses during interpersonal stress, providing a plausible psychological link between greater ACE exposure and higher CA (74). University-student studies have reported negative associations between ACEs or childhood maltreatment and PR and statistical indirect associations involving PR (36, 37, 75), while Chinese college-student research has linked childhood trauma and lower resilience with higher aggression-related outcomes (37, 76). In cyber contexts, lower resilience has also been associated with cyberbullying perpetration and risky online behavior (38, 39, 77); broader evidence on positive developmental experiences and resilience-related assets similarly links protective resources with more favorable post-adversity adjustment (78). Thus, the mediation analysis identified a statistical indirect pathway in which higher continuous ACE scores were associated with lower PR, and lower PR was associated with higher CA. Because the data are cross-sectional, this indirect effect does not establish temporal ordering or a causal mechanism; longitudinal research is needed to test whether changes in PR prospectively account for the association between ACE exposure and subsequent CA.

### 4.3 Moderating role of physical exercise

Among college students, PE moderated the second-stage pathway of the statistical mediation model, specifically the association between PR and CA. PE may strengthen the protective association of PR with lower levels of CA. This interpretation is consistent with evidence that higher physical activity is associated with stronger self-regulatory resources and lower aggressive behavior among college students (42). As an important means of promoting physical and psychological well-being, PE may enhance positive emotional experiences and reduce negative affect (79, 80). One possible interpretation, consistent with prior research, is that regular exercise is associated with more adaptive emotion regulation and lower susceptibility to stress-related negative affect (81). When college students have experienced adverse events such as psychological maltreatment, those with limited psychological resources, including lower PR, may be more vulnerable to anger and other negative emotions (82). These emotional responses may, in turn, be associated with a greater likelihood of CA (83). In contrast, individuals who regularly participate in PE may be better able to regulate their psychological states, and this enhanced self-regulatory capacity may be associated with a lower likelihood of CA (84).Previous research has also shown that PE may contribute to higher levels of PR. Individuals with greater PR are generally better able to cope with difficulties and setbacks and may be less vulnerable to adverse psychological outcomes, including anxiety, depression, and CA (85–88). The observed moderating effect therefore supports further investigation of PE as a potentially modifiable correlate in interventions targeting CA among college students. Encouraging students to participate in diverse and appropriately structured physical activities, including supervised competitive sports, may promote PR and contribute to lower levels of CA among students with histories of adversity (89).

## 5 Research contributions

The present study extends the cumulative risk model to the study of CA among college students. Rather than examining only the general association between ACEs and CA, it further evaluates the nonlinear pattern associated with the cumulative ACE count. The findings indicate that the association between the cumulative ACE count and CA is nonlinear, with the significant positive quadratic term supporting an overall upward-curving pattern. This finding moves beyond the predominant focus of previous research on single adverse experiences or simple linear associations and broadens the applicability of the cumulative risk model to online behavioral outcomes. In addition, by incorporating PR and PE into a unified analytical framework, the study shows that PR statistically mediates the association between the continuous ACE score and CA, whereas PE moderates the association between PR and CA. Together, these findings support an explanatory framework of “risk accumulation–psychological resources–behavioral regulation” and provide a more integrated theoretical perspective on how early-life risk, individual protective resources, and health-related behaviors are jointly associated with CA among college students.

The present study provides an empirical basis for the stratified identification and comprehensive prevention of CA in higher education settings. First, college students with higher levels of cumulative ACE exposure may warrant particular attention in risk assessment and support services. Subject to the principles of privacy protection and voluntary participation, university mental health and student support programs could place greater emphasis on assessing emotion-regulation difficulties, hostile cognitive tendencies, and risks related to online behavior in this group (90). Second, although ACEs represent historical risk factors that cannot be altered retrospectively, PR and PE are comparatively modifiable. Universities could therefore integrate resilience training, stress management, emotion-regulation education, and regular physical activity through courses, group-based counseling, and campus sport programs to strengthen students’ capacity to cope with adversity and regulate negative emotions (91–93). Compared with approaches that focus only on managing CA after it occurs, a comprehensive strategy that combines early risk identification, the development of psychological resources, and the promotion of PE may contribute more effectively to a preventive support system for CA among college students.

## 6 Limitations and future directions

Several limitations should be acknowledged. First, the present study employed a cross-sectional design. Although this design allowed us to identify patterns of association among ACEs, PR, PE, and CA and to test statistical mediation and moderation, it did not permit conclusions regarding temporal ordering or causal direction. Accordingly, the findings should not be interpreted as indicating that ACEs inevitably lead to CA or that PR and PE exert direct causal protective effects. Future research should adopt longitudinal follow-up designs, cross-lagged models, or intervention studies to examine the dynamic relationships among these variables and clarify their potential causal mechanisms. Second, the data were primarily derived from self-report questionnaires, and the assessment of ACEs relied on retrospective recall. The findings may therefore have been affected by recall bias, social desirability, and common method bias. Future studies could incorporate multi-source data, such as parent or peer reports, records of online behavior, interview data, and objective indicators of physical activity, to improve the reliability of the findings.

Third, purposive sampling was used in the present study. Although the sample included students from multiple provinces and different types of universities, it may not fully represent the broader population of Chinese college students. The generalizability of the findings across regions, institutional types, and cultural contexts therefore requires further examination. In addition, the final de-identified dataset did not retain province- or university-level identifiers, which limited a more detailed description of the post-screening sample distribution across recruitment sites. Future research could employ stratified random sampling, recruit participants from a wider range of settings, and compare associations across sex, year of study, urban–rural household registration, and regional groups. In addition, the present study assessed cumulative risk primarily by counting exposure across five categories of ACEs. Although this approach captures the overall burden of adversity, it may obscure differences in the type, severity, and configuration of adverse experiences. Future studies could further examine the independent and interactive associations of emotional abuse, physical abuse, sexual abuse, emotional neglect, and physical neglect with CA, and could use latent class analysis or network analysis to identify more specific risk profiles. Finally, the present study focused only on the statistical mediating role of PR and the moderating role of PE. Future research could extend the current model by incorporating additional psychosocial factors, such as social support, emotion regulation, and hostile attribution bias, to develop a more comprehensive explanatory framework.

## 7 Conclusion

Drawing on the cumulative risk model, the present study examined the associations among ACEs, PR, PE, and CA among college students. The findings showed that a higher cumulative ACE count was significantly associated with higher levels of CA, and the significant positive quadratic term supported an overall upward-curving association. PR statistically mediated the association between the continuous ACE score and CA, while PE significantly moderated the association between PR and CA; specifically, higher levels of PE strengthened the inverse association between PR and CA. Overall, the findings suggest that ACEs represent an important risk-related correlate of CA among college students, whereas PR and PE may serve as potentially modifiable protective resources. Universities may therefore consider integrating early risk identification, resilience-building programs, emotion-regulation education, and regular physical activity into comprehensive strategies for the prevention and intervention of CA. Given the cross-sectional design, however, these findings should be interpreted as statistical associations rather than causal relationships.

## Data Availability

The original contributions presented in the study are included in the article/supplementary material. Further inquiries can be directed to the corresponding author.
